# Depressive symptoms and anger and aggression in Russian adolescents

**DOI:** 10.1186/s13034-023-00677-w

**Published:** 2023-11-16

**Authors:** Vladislav Ruchkin, Andrew Stickley, Roman Koposov, Denis G. Sukhodolsky, Johan Isaksson

**Affiliations:** 1 Child and Adolescent Psychiatry Unit, Department of Medical Sciences, Uppsala University, Uppsala, S-751 85 Sweden; 2 Child Study Center, Yale University Medical School, New Haven, CT 06520 USA; 3 Department for Preventive Intervetion for Psychiatric Disorders, National Center of Neurology and Psychiatry, Kodaira, Tokyo Japan; 4 Regional Centre for Child and Youth Mental Health and Child Welfare, Northern Norway, UiT The Arctic University of Norway, Tromsø, Norway; 5 Department of Epidemiology and Modern Technologies of Vaccination, Sechenov First Moscow State Medical University, Moscow, Russia; 6 Center of Neurodevelopmental Disorders (KIND), Centre for Psychiatry Research, Department of Women’s and Children’s Health, Karolinska Institutet & Stockholm Health Care Services, Region Stockholm, Stockholm, Sweden

**Keywords:** Major depression, Subthreshold depressive symptoms, Comorbidity, Aggression, Anger, Adolescents, Gender

## Abstract

Research among adolescents exploring the association between depressive symptoms and aggression has produced inconsistent findings. This study investigated the prevalence of clinically significant (current major depressive episode) and subthreshold depressive symptoms in a general population sample of adolescents from Northern Russia and explored their association with aggression and anger, while controlling for comorbid mental health problems. The sample consisted of 2600 participants, aged 13–17 years (59.5% female; 95.7% ethnic Russian). Symptoms of a current major depressive episode, types of anger and aggression (anger rumination, trait anger, physical, verbal and social aggression) and comorbid problems (posttraumatic stress, alcohol use, anxiety, and hyperactivity/impulsivity) were assessed by means of self-reports. The prevalence of a clinically significant depressive episode in the past month was 3.5%, while for subthreshold depression it was 21.6%. All anger and aggression variables, as well as comorbid problems increased together with increasing levels of depression. The association between overt aggressive behavior and depression was primarily explained by comorbid mental health problems, whereas anger rumination and social aggression had more direct associations with depression, independent of comorbidity. Among adolescents with depression, boys reported higher levels of social and verbal aggression and of anger rumination than girls. The results of this study suggest that interventions aiming to reduce aggressive behavior in adolescents should consider depression and its comorbid conditions.

## Introduction

Research suggests that individuals suffering from psychiatric disorders have an increased risk of engaging in aggressive behavior [1–3] and most researchers have concurred that a modest but statistically significant relationship exists between violence and severe mental illness [4]. Among the disorders that are likely to increase the risk for aggressive behavior previous research has indicated schizophrenia [5], alcohol and drug use [6], posttraumatic stress disorder [7], attention-deficit/hyperactivity disorder (ADHD) [8], and autism spectrum disorder [9]. Despite seeming less intuitive, an association has also been consistently reported between depression and aggressive behavior both in adults [e.g. 10], and in children and adolescents [11, 12], in clinical and general population samples.

Depression is one of the most common psychiatric disorders, with its prevalence rates rising substantially throughout adolescence. Thus, Costello et al. [13] reported that the overall prevalence of any depressive disorder was 2.8% in children under 13 years of age and 5.6% in adolescents aged 13 to 18. Other studies have reported higher numbers, including 10.5% for any depressive disorder and 29.2% for subthreshold depression in a large European study of adolescents [14], with a 25% lifetime prevalence of major depressive disorder by the end of adolescence [15].

Historically, the relation between depression and aggression was thought to reflect a broader association between internalizing and externalizing forms of psychopathology [16] and hence, could be explained by comorbid conditions rather than by the depression itself [17]. Indeed, considerable research has shown an association between aggression and anxiety, ADHD, substance abuse and posttraumatic stress [18, 19], which are all also highly comorbid with depression. However, a recent study of adults from the general population that controlled for potential confounders, such as comorbid alcohol and drug use, familial confounding, a previous history of violence and sociodemographic factors, was still able to demonstrate a two- to three-fold increase in the risk of committing a violent crime by depressed individuals [10]. Similarly, a study of juvenile offenders showed an independent effect of depression on aggressive behavior [12].

From a theoretical standpoint, several overlapping models have been proposed to explain the association between depressive symptoms and aggression (for a review see [20]). Specifically, it has been suggested that some depressive symptoms, such as irritability, may be expressed behaviorally in interpersonal relationships through aggression and rule breaking (the acting out model), and this can be exacerbated over time both through the youth’s failure in their relations with individuals in the surrounding social environment caused by aggressive behaviors (the failure model), and through the escalating reciprocal reinforcement of depressive symptoms and aggression, which is further impacted by other nonspecific risk factors, such as comorbidity (the reciprocal or shared risk factor model). Indeed, as depressive symptoms may lead to impaired neurocognitive performance [21], problems with emotional regulation and poor impulse control [22], and increased irritability and anger [23], it seems reasonable to expect that some specific symptoms of depression may potentially increase the likelihood of aggressive behavior. For example, it has been suggested that it may not be depressed mood itself, but rather specific depressive symptoms, such as agitation [24] or psychotic features [25] that increase the risk for aggression. Similarly, given that aggression is a very heterogeneous behavior in its own right, depression may affect cognitive, emotional and behavioral facets of aggression. For example, anger rumination is a cognitive-emotional precursor of aggression [26], referring to the tendency to dwell on frustrating experiences and recall past anger experiences [27], that has been closely related to and often co-occurs with sadness [28]. Trait anger represents a disposition to perceive situations as annoying or frustrating, and the tendency to react in such situations with more frequent elevations in state anger [29], and it has been connected to depression both through emotion regulation and anger rumination [30]. Finally, the behavioral expressions of anger can take the form of physical or verbal acts [31], or more subtle forms, such as social aggression, which refers to behaviors that intentionally damage interpersonal relationships and/or social status through non-confrontational and concealed methods [32], and all these forms of aggression have been similarly linked to the cognitive-emotional aspects of depression [33].

Previous research has suggested that there are substantial gender differences in the prevalence of both depression and aggression. While overall depression rates are generally similar by gender in childhood [e.g. 13], females have been consistently found to have an increased risk of depression during adolescence [34]. In contrast, research on aggression has shown that boys consistently report more overt aggression compared to girls [35, 36], while in a large international study of children [37] boys reported being more physically aggressive than girls across all countries, but no consistent gender differences were observed in social aggression, particularly in studies from non-Western countries. Other studies have suggested however, that gender differences in social aggression may become more apparent in adolescence, with a particular increase in girls [38]. Despite these observed gender differences, until now, findings regarding the strength of the association between depression and aggression in boys versus girls have been inconsistent, suggesting no gender differences [11], or a stronger association in males [39, 40] or females [41], thus highlighting the need for more research.

Hence, considering the potential complexity of the association between depression, its comorbid conditions and different types of aggression, when examining the association between depression and aggression it is important to (1) clearly define the depressive condition, (2) evaluate different types of aggression, (3) control for a broad range of comorbid psychopathology, which by itself can potentially impact aggressive behavior, and (4) take into account the potential gender differences. The aim of the current study was therefore to (1) assess the prevalence of depressive symptoms corresponding to a major depressive episode and subthreshold depression in a general population sample of 13–17 year old adolescents from Northern Russia; (2) investigate the associations between a major depressive episode, subthreshold depression, and different types of anger and aggression (anger rumination, trait anger, and verbal, physical and social aggression), and comorbid problems (posttraumatic stress, alcohol use, anxiety, hyperactivity/impulsivity); while (3) exploring the role of gender differences in these associations.

## Methods

### Study population

This study was conducted in Arkhangelsk, the largest city in the Northern European part of Russia. According to census data [42], the population of Arkhangelsk is slightly over 349,000 and approximately 30,000 adolescents are in the 13–17-year-old age range. The socioeconomic status of the population is generally in the low to average range for Russia. Permission to conduct this study in selected schools was obtained from the Arkhangelsk city administration and the study was conducted in collaboration with the local schools’ administration. The study involved all the main districts of the city and the number of potential participants from each district was calculated in proportion to the total number of adolescents of the relevant age residing in the district. A randomized selection procedure was used to obtain a representative sample with school buildings and classes designated as the units of randomization. In stage one, 14 schools were randomly selected from 71 eligible schools, all of which agreed to participate and were included in the study, yielding a total of 210 classes with students of the relevant age. In stage two, data were collected from students in 70 randomly selected classes (out of 210), resulting in a sample of 2838 students. Students with incomplete reports (missing responses) were excluded. Adolescents from the excluded group (n = 238) were more likely to be male (59.1% vs. 40.9%, χ^2^(1) = 30.66, p < .001), and reported higher levels of verbal (M(SD) = 5.00(4.32) vs. 4.26(3.52); t = 2.87; p < .001, physical (M(SD) = 4.14(4.02) vs. 2.97(3.39); t = 4.77; p < .001) and social aggression (M(SD) = 16.06(6.77) vs. 15.03(4.65); t = 2.96; p < .001). The excluded adolescents did not differ from those in the study on any other variables.

The final sample consisted of 2600 participants, who ranged in age from 13 to 17 years old (M(SD) = 14.90(1.13)). The composition of the sample was 59.5% female (n = 1547) and predominantly of Russian origin (95.7%), followed by a small proportion of other (predominantly Slavic) nationalities, an accurate reflection of the local public school population. Most of the participants (75.6%) came from two-parent families, whereas 24.4% had divorced, separated or widowed parents. According to student reports, 93.0% of their fathers and 94.4% of mothers had completed the equivalent of a high school education or beyond.

### Procedure

Parents were informed of the survey prior to the study and were offered the opportunity to decline participation. Before the survey’s administration, students were read a detailed assent form describing their participation and confidentiality, and were asked to sign it to indicate assent. Students also had the option to decline to participate at the time of administration (parent and student refusals were less than 1%). Students completed the survey in one class period during a normal school day. Surveys were administered in Russian. This study was approved by the institutional ethics committee and scientific council of the Institute of Psychiatry, Northern State Medical University (Arkhangelsk, Russia) (decision from 2003-02-14) and permission to conduct the study was obtained from the Arkhangelsk city administration.

## Measures

### Depressive symptoms

*Depressive symptoms* were measured with an adapted version of the Center for Epidemiologic Studies-Depression Scale (CES-D) [43], which has excellent psychometric properties with adolescents [44], and has been used with Russian adolescents previously [45]. Respondents were asked to indicate the presence of ten depressive symptoms during the past 30 days on a three-point scale: “Not true” (0); “Somewhat true” (1); or “Certainly true” (2). The scale had a good internal consistency – Cronbach’s α was 0.84.

Based on the CES-D items, a proxy for a current major depressive episode was created following the Diagnostic and Statistical Manual of Mental Disorders, Fifth Edition (DSM-5) criteria [46], according to which, five or more symptoms of depression should be present, and at least one of these symptoms should include either (1) depressed mood, or (2) loss of interest. *Depressed mood* in the past 30 days was determined based on a positive response (Certainly true) to one or more of the following four items: “I felt really down”; “I felt like crying”; “I felt that I could not shake off my sad feelings even with help from my family or friends”; and “I felt bothered by people and things” (irritable mood). *Diminished interest* was assessed with the item: “I have lost my interest in other people or things”. *Other symptoms of depression* used to create a proxy for a current major depressive episode included decreased appetite (“I did not feel like eating; my appetite was poor”); problems with sleep (“I didn’t sleep well”); loss of energy (“I was tired”); feelings of excessive guilt (“I felt that many bad things were my fault”); and diminished ability to think or concentrate (“I found it difficult to concentrate”). Four or more of these symptoms (all with “Certainly true” scores), in addition to either depressed mood or diminished interest, had to be present to indicate a current major depressive episode. Those, who fulfilled the above criteria, were denoted as having current clinically significant depression (CSD), and coded as 2. Those students who reported the presence of the same criteria, but scored some of the items as Somewhat true, were denoted as having a subthreshold depression score (SDS), and coded as 1. Finally, all other students whose symptoms did not fulfill the criteria for either CSD or SDS were considered as having a low depression score (LDS), and coded as 0.

### Anger and aggression

*The Anger Rumination Scale* (ARS) [27] measures the tendency to focus attention on angry moods, recall past anger experiences, and think about the causes and consequences of anger episodes. The Russian version of the ARS is comprised of 17 items (e.g. “When someone makes me angry I can’t stop thinking about how to get back at them”, “After an argument is over, I keep fighting with this person in my imagination”, and “I think about the reasons people treat me badly”), rated on a 4-point scale from “Almost never” (1) to “Almost always” (4). The total score can range from 17 to 68 with higher scores indicating more anger rumination. The ARS has demonstrated adequate internal consistency and test–retest reliability as well as convergent and discriminant validity [27]. Cronbach’s α for the scale in this study was 0.95.

*The Trait Anger Scale* of the State Trait Anger Expression Inventory (STAXI) [29] represents one of the most commonly used measures of anger and consists of 10 items that assess a general tendency to experience anger, either in the absence of a direct provocation or as a result of specific triggers, such as criticism or unfair treatment by others (e.g. “I feel infuriated when I do a good job and get a poor evaluation” and “I get angry when I’m slowed down by others’ mistakes”). Items are rated on a 4-point scale (from “Almost never” (1) to “Almost always” (4)). The total score can range from 10 to 40 with higher scores indicating more trait anger. The scale has been validated in Russia previously [47]. Cronbach’s α for the scale was 0.92.

*Physical and verbal aggressive behaviors* [48] were assessed with 5 items each, describing common forms of aggressive behaviors in children and adolescents (e.g. “Pushed or shoved somebody”, and “Punched someone in a fight” for physical aggression and “Teased others” and “Called others names” for verbal aggression). The students rated the frequency of their aggressive behavior in the past 30 days on a 4-point scale (from “Never” (0) to “5 or more times” (3)). The possible total score could range from 0 to 15 for each scale, with higher scores indicating more aggression. These scales have demonstrated good psychometric properties with Russian adolescents previously [49]. Cronbach’s alphas for the scales were respectively 0.80 and 0.82.

*Social aggression* [48] was assessed with 9 items describing non-confrontational behaviors aimed at intentionally damaging social relationships, status and reputation directed towards a peer. Specifically, the scale asked the respondents to think about a peer they “did not like very much” and answer if they have ever engaged in behavior such as “Spread rumors/gossip about this person”, “Told others not to be friends with this person”, and “Told others bad things about this person”). The students rated each item using a 4-point scale with response options ranging from “Almost never” (1) to “Almost always” (4). The possible total score can range from 9 to 36 with higher scores indicating more social aggression. The construct of social aggression is distinct from the measures of physical and verbal aggression and the scale has also previously shown good psychometric properties with Russian adolescents [49]. Cronbach’s α for the scale in this study was 0.83.

### Comorbid conditions

*Posttraumatic stress* was assessed with the Child Post-Traumatic Stress - Reaction Index (CPTS-RI) [50], a 20-item scale assessing the frequency of posttraumatic symptoms (e.g., “Do you get scared or afraid because you think about bad things that have happened to you?,” “Do thoughts or pictures of bad things that have happened to you come back to you, even when you don’t want them to?,” “If someone comes up behind you all of a sudden, or if you hear a loud noise, do you jump?”) on a 5-point scale, ranging from “Never” (0) to ”Most of the time” (4). The degree of reaction is categorized as doubtful (score < 12), mild (score = 12–24), moderate (score = 25–39), severe (score = 40–59), or very severe (score ≥ 60). Total scores can range from 0 to 80, with higher scores corresponding closely with a clinical diagnosis of posttraumatic stress disorder (PTSD) [51], including in Russian adolescents [52]. Cronbach’s α = 0.87.

*Alcohol use* was assessed with three items derived from the Monitoring the Future Scale [53]. Students responded if they had had a drink of beer, wine or strong alcohol (separate items for each type of drink) in the past 30 days on a four-point scale, ranging from “Never” (0) to “More than a few times” (3). The possible scale score ranges from 0 to 9, with higher scores indicating greater alcohol use.

The *hyperactivity/inattention scale* of the Strengths and Difficulties Questionnaire (SDQ) [54] was used to assess these symptoms. The SDQ represents one of the most widely used screening instruments of mental health problems in children and adolescents in international settings [see e.g. 55], and has been previously validated with Russian adolescents [56]. It consists of five items (e.g., ”I am restless. I cannot stay still for long”, “I am constantly fidgeting or squirming” and “I am easily distracted”). Respondents are asked to rate their behavior and feelings for the past 6 months on a three-point scale (from “Not True” (0) to “Certainly True” (2)). The total score could range from 0 to 10 with higher scores indicating more hyperactivity/inattention symptoms. Cronbach’s α = 0.65.

*Anxiety symptoms* were measured with a 12-item scale [48], inquiring about worrisome thoughts and feelings (e.g. “I feel nervous when I get called on in class”, “I stay away from things that make me nervous”), that are rated on a three-point scale (from “Not True” (0) to” Certainly True” (2)). This measure has been extensively used with Russian adolescents previously [e.g. 57]. The total score ranged from 0 to 24, with higher scores reflecting increasing anxiety symptoms. Cronbach’s α = 0.83.

A proxy for *Socioeconomic Status (SES)* was created using students’ reports on a single-parent family status (1/0), a lower level of parental education (incomplete college education or lower, 1/0), and parental employment status (full time (0), part time (1) and unemployed (2)). The possible total score could range from 0 to 6 with higher scores indicating lower SES.

### Statistical analyses

Data were analyzed using SPSS version 28.0. Chi-square and independent sample t-tests were used for univariate comparisons of demographic characteristics and of dependent variables across gender. General linear models (GLM) multivariate analysis of covariance (MANCOVA) was used to determine main and interaction effects across the three fixed factors of depression (LDS = 0, SDS = 1, CSD = 2) and gender (boys = 1, girls = 0), while adjusting for the covariates of age and SES. In the first set of analyses, the dependent variables included anger rumination, trait anger, physical, verbal and social aggression, together with the comorbid problems of posttraumatic stress, anxiety, hyperactivity/inattention and alcohol use, in order to investigate the associations between depression and comorbidity and to explore if such associations would be found in the study population. The MANCOVA was subsequently repeated, adjusting for the comorbid symptoms of posttraumatic stress, alcohol use, anxiety and hyperactivity/inattention (instead of using them as dependent variables), in addition to the covariates of age and SES.

The unique contributions of each of the two fixed factors (depression and gender), of one interaction term, and of the covariates were assessed through follow-up between-subject tests and unstandardized parameter estimates derived from the MANCOVA. Results are presented as means (M) and standard deviations (SD), and for individual outcomes, as partial eta squared (η^2^), a common metric of effect size that represents the unique amount of variance explained by each predictor variable. In the analyses, two-tailed tests with a p-value of < 0.05 were considered statistically significant. In order to avoid Type I errors, the p-values have been adjusted to account for multiple testing.

## Results

### Prevalence of individual depressive symptoms, subthreshold depression and a major depressive episode by gender

The prevalence of depressive symptoms, constituting a major depressive episode, is presented in Table 1 (“Certainly true” scores only), listed according to the DSM-5 criteria for a current major depressive episode.

**Table 1:** Prevalence of depressive symptoms (certainly true scores only) constituting a current major depressive episode in boys and girls

Symptoms	Boys	Girls	Chi-square, p
**Depressed mood (one of the following)**:	252 (24.7%)	545 (35.9%)	35.55; <0.001
I felt really down	89 (8.4%)	214 (13.8%)	18.30; <0.001
I felt like crying	58 (5.5%)	267 (17.3%)	80.90; <0.001
I felt that I could not shake off my sad feelings even with help from my family or friends	69 (6.5%)	154 (9.9%)	9.78; <0.01
I felt bothered by people and things	132 (12.4%)	247 (16.0%)	6.83; <0.01
**Diminished interest**:			
I have lost my interest in other people or things	98 (9.4%)	139 (9.1%)	0.01; ns
**Other depressive symptoms (≥ 4 symptoms present)**:	47 (4.5%)	72 (4.7%)	0.06; ns
I did not feel like eating; my appetite was poor	72 (6.8%)	119 (7.7%)	0.77; ns
I didn’t sleep well	136 (12.7%)	149 (9.6%)	6.23; <0.05
I was tired	258 (24.2%)	424 (27.4%)	3.36; ns
I felt that many bad things were my fault	86 (8.1%)	164 (10.6%)	4.85; <0.05
I found it difficult to concentrate.	178 (16.7%)	266 (17.2%)	0.12; ns

The prevalence of *depressed mood* during the past 30 days was significantly higher in girls for all corresponding symptoms, whereas no gender differences were found with regard to *diminished interest*. In addition, several *other depressive symptoms* were evaluated, including decreased appetite, problems with sleep, loss of energy, feelings of inappropriate guilt, and diminished ability to concentrate, and no gender differences were found in the combined prevalence of the 4 (or more) of these symptoms that had to be present to indicate a current major depressive episode. A total of 92 (3.5%) adolescents were identified as having clinically significant depressive symptoms (corresponding to a current *major depressive episode* with the symptoms present in the past 30 days), denoted as a CSD. In addition, 561 (21.6%) adolescents reported the presence of the same depressive symptoms, but scored some of the symptoms as “Sometimes true”, and were considered as having a SDS. All other adolescents (n = 1947; 74.9%) were considered as having LDS. No gender differences were observed, when comparing the CSD rates (35 (3.3%) in boys vs. 57 (3.7%) in girls, χ^2^ = 0.24; Cramer’s V = 0.010; ns), but when the SDS rates in adolescents were compared, the prevalence of depression became significantly more prevalent in girls (184 /17.5%) in boys vs. 377 (24.4%) in girls, χ^2^ = 18.44; Cramer’s V = 0.084; p < .001).

### Generalized linear modeling

Tables 2 and 3 present the results of comparisons of the aggression scores by gender and depression.

**Table 2:** Types of anger and aggression and comorbid problems (M (SD)) in relation to different depression scores in boys (B) and girls (G)

		LDS	SDS	CSD	Total group
Physical aggression	B	4.17 (3.82)	4.81 (3.77)	6.17 (5.19)	4.34 (3.88)
	G	1.87 (2.52)	2.41 (2.88)	2.84 (2.74)	2.03 (2.63)
Verbal aggression	B	4.61 (4.00)	5.86 (3.61)	6.69 (4.90)	4.90 (4.00)
	G	3.60 (3.00)	4.40 (3.21)	4.65 (2.93)	3.83 (3.07)
Social aggression	B	14.61 (4.40)	15.86 (5.50)	20.31 (10.26)	15.02 (5.02)
	G	14.89 (4.32)	15.22 (4.22)	16.77 (6.07)	15.04 (4.39)
Anger rumination	B	28.89 (10.09)	33.41 (10.07)	42.40 (15.53)	30.13 (10.68)
	G	31.06 (9.17)	35.23 (9.64)	38.61 (9.97)	32.35 (9.81)
Trait anger	B	19.33 (6.60)	22.01 (6.44)	25.46 (8.89)	20.00 (6.81)
	G	20.89 (6.56)	23.67 (6.94)	26.84 (7.54)	21.79 (6.87)
Alcohol use	B	4.37 (3.10)	4.79 (3.03)	4.09 (3.71)	4.43 (3.11)
	G	4.22 (2.98)	4.81 (2.95)	5.40 (2.82)	4.41 (2.98)
Posttraumatic stress	B	16.03 (10.02)	23.64 (12.22)	30.71 (16.07)	17.84 (11.30)
	G	19.11 (10.03)	26.08 (11.60)	35.86 (14.05)	21.43 (11.36)
Hyperactivity/	B	3.75 (1.95)	4.76 (2.00)	6.07 (1.68)	4.08 (2.04)
impulsivity	G	3.39 (1.97)	4.66 (1.82)	5.31 (2.13)	3.67 (2.03)
Anxiety	B	11.77 (5.64)	14.36 (5.79)	13.91 (6.87)	12.30 (5.80)
	G	13.62 (5.53)	14.92 (5.24)	16.98 (4.86)	14.06 (5.49)

*Note*: M(SD) – Mean (Standard Deviation); LDS – low depression score, SDS – subthreshold depression score, CSD – clinically significant depression (corresponding to DSM-V criteria for a current major depressive episode)

**Table 3:** Effect sizes for the associations between depression, gender, comorbid problems and different types of anger and aggression (η^2^, p)

	Age	SES	Gender	Depression	Depression x Gender
Physical aggression	0.003, < 0.01	0.001, ns	**0.040, < 0.001**	**0.011, < 0.001**	0.001, ns
Verbal aggression	0.000, ns	0.000, ns	**0.012, < 0.001**	**0.018, < 0.001**	0.001, ns
Social aggression	0.002, < 0.05	0.001, ns	0.000, ns	**0.023, < 0.001**	**0.006, < 0.001**
Anger rumination	0.002, < 0.05	0.000, ns	**0.005, < 0.001**	**0.055, < 0.001**	0.003, < 0.01
Trait anger	0.001, ns	0.000, ns	0.003, < 0.01	**0.044, < 0.001**	0.000, ns
Alcohol use	**0.096, < 0.001**	0.000, ns	0.000, ns	0.000, ns	0.002, < 0.05
Posttraumatic stress	0.000, ns	**0.006, < 0.001**	**0.007, < 0.001**	**0.112, < 0.001**	0.001, ns
Hyperactivity	0.002, < 0.05	0.002, < 0.05	0.003, < 0.01	**0.074, < 0.001**	0.001, ns
Anxiety	0.002, < 0.05	0.002, < 0.05	**0.007, < 0.001**	**0.024, < 0.001**	0.003, < 0.05

*Note*: SES - proxy for socioeconomic status. η^2^ - Eta-squared, p – significance level. Adjusted p-values (that were statistically significant after multiple testing) are marked in bold

The results suggest that the proposed model was significant and could explain 16% of the variance in the outcome variables (Wilks’ lambda = 0.836; F (9, 2584) = 56.21, p < .001, η2 = 0.164). The main effect for Depression was significant (Wilks’ lambda = 0.832; F (18, 5168) = 27.62, p < .001, η2 = 0.088), suggesting differences in the levels of aggression and comorbid problems by depression. The follow-up tests (Table 3) indicated that all aggression variables, as well as posttraumatic stress, anxiety and hyperactivity/inattention levels increased together with increasing levels of depression from LDS to SDS to CSD. The main effect for Gender was also significant (Wilks’ lambda = 0.926; F (9, 2584) = 23.10, p < .001, η2 = 0.074), and the follow-up tests demonstrated significant gender differences in all dependent variables, except for social aggression and alcohol use, with boys having higher ratings on physical and verbal aggression and hyperactivity/inattention, but lower levels of anger rumination, trait anger, posttraumatic stress and anxiety. The main effect for Age was significant (Wilks’ lambda = 0.885; F (9, 2584) = 37.24, p < .001, η2 = 0.115), with the follow-up tests demonstrating a decrease in physical aggression, but an increase in anger rumination, social aggression, alcohol use, hyperactivity/inattention and anxiety with increasing age. The main effect for SES was significant (Wilks’ lambda = 0.990; F (9, 2584) = 3.02, < 0.001, η2 = 0.010), and yet the follow-up tests suggested that none of the aggression variables were related to SES and the differences were explained by posttraumatic stress, hyperactivity/inattention and anxiety. Finally, the interaction effect for Depression x Gender was significant (Wilks’ lambda = 0.978; F (18, 5168) = 3.19, p < .001, η2 = 0.011), indicating gender-specific differences in the dependent variables in relation to depression, and more specifically in social aggression and anger rumination (higher in boys with depression), as well as in anxiety and alcohol use (higher in girls with depression) (Table 3).

Table 4 presents the individual effects for each dependent variable, using the same model as above, but now also adjusting for comorbid problems (posttraumatic stress, alcohol use, anxiety and hyperactivity/inattention), in addition to the previously used covariates of age and SES.

**Table 4:** Effect sizes for each dependent variable (types of aggression, anger rumination, trait anger) (η^2^, p) after adjusting for comorbidity

	Effects for covariates		Effects for main variables			Effects for comorbidity			
	Age	SES	Gender	Depression	Depression x Gender	PTS	Alcohol use	Hyperactivity/ inattention	Anxiety
Physical aggression	**0.018, < 0.001**	0.000, ns	**0.051, < 0.001**	0.000, ns	0.002, ns	**0.023, < 0.001**	**0.034, < 0.001**	**0.028, < 0.001**	**0.011, < 0.001**
Verbal aggression	**0.006, < 0.001**	0.000, ns	**0.020, < 0.001**	0.001, ns	0.003, < 0.05	**0.018, < 0.001**	**0.060, < 0.001**	**0.045, < 0.001**	0.002, < 0.05
Social aggression	0.001, ns	0.000, ns	**0.012, < 0.001**	.**006, < 0.001**	**0.010, < 0.001**	**0.045, < 0.001**	**0.029, < 0.001**	**0.029, < 0.001**	0.001, ns
Anger rumination	0.001, ns	0.000, ns	0.004, < 0.01	0.004, < 0.01	**0.007, < 0.001**	**0.119, < 0.001**	**0.009, < 0.001**	**0.010, < 0.001**	**0.074, < 0.001**
Trait anger	0.001, ns	0.000, ns	0.000, ns	0.002, ns	0.001, ns	**0.032, < 0.001**	**0.034, < 0.001**	**0.094, < 0.001**	**0.025, < 0.001**

*Note*: SES - proxy for socioeconomic status; PTS- posttraumatic stress, η^2^ - Eta-squared, p – significance level. Adjusted p-values (that were statistically significant after multiple testing) are marked in bold

The results suggest that the proposed model was significant and explained 6.5% of the variance in the outcome variables (Wilks’ lambda = 0.935; F (5, 2584) = 36.17, p < .001, η2 = 0.065). The main effect for Depression was significant (Wilks’ lambda = 0.986; F (10, 5168) = 3.55, p < .001, η2 = 0.007), suggesting remaining differences in aggression in relation to depression. The follow-up tests (Table 4) indicated that depressed adolescents now differed from others only with regard to social aggression and anger rumination. The main effect for Gender was also significant (Wilks’ lambda = 0.938; F (5, 2584) = 34.20, p < .001, η2 = 0.062), and the follow-up tests showed significant differences between boys and girls in all aggression variables (except for trait anger), with higher levels in boys. The main effect for Age was significant (Wilks’ lambda = 0.980; F (5, 2584) = 10.52, p < .001, η2 = 0.020), with the follow-up tests demonstrating a decrease in physical, but an increase in verbal aggression with increasing age. The main effect for SES was not significant (Wilks’ lambda = 0.997; F (5, 2584) = 1.42, ns, η2 = 0.003). Finally, the main effects for posttraumatic stress (Wilks’ lambda = 0.856; F (5, 2584) = 86.69; p < .001, η2 = 0.144), alcohol use (Wilks’ lambda = 0.919; F (5, 2584) = 45.31; p < .001, η2 = 0.081), hyperactivity/inattention (Wilks’ lambda = 0.887; F (5, 2584) = 66.14, p < .001, η2 = 0.113) and anxiety (Wilks’ lambda = 0.899; F (5, 2584) = 58.17, p < .001, η2 = 0.101) were all significant, with increasing levels of comorbid problems being related to all of the aggression variables, except for anxiety, which was related to increased levels of anger rumination and trait anger, but was not related to social aggression and was related to lower levels of physical and verbal aggression. The interaction effect for Depression x Gender was also significant (Wilks’ lambda = 0.984; F (10, 5168) = 4.22, p < .001, η2 = 0.008), indicating gender-specific differences in aggression in relation to depression (Table 4), with higher levels of social and verbal aggression and of anger rumination in depressed boys, as compared to depressed girls.

In order to avoid Type I errors, the p-values have been adjusted for multiple testing (x45) and hence only p-values of 0.0011 and lower were considered statistically significant. These values have been marked in bold font in both Tables 3 and 4.

## Discussion

This study found that the prevalence of current CSD in Russian adolescents aged 13–17 years old in the general population (3.5%) corresponding to a current major depressive episode in the past month was similar to that observed in adolescents in other countries. Levels of all the aggression variables, as well as posttraumatic stress, and anxiety and hyperactivity/inattention increased together with increasing levels of depressive symptoms, while all of the comorbid conditions were also associated with aggression. When adjusting for comorbidity, only the attenuated association between depressive symptoms and social aggression and anger rumination remained statistically significant. Depressed boys, as compared to depressed girls, reported higher levels of social and verbal aggression and of anger rumination.

To our knowledge, this is the first study to report on the prevalence of major depression in Russian adolescents. A recent report from the World Health Organization [58] found that the prevalence of depression in the Russian general population was 5.5%, although without reference to any specific age groups. While the prevalence of individual depressive symptoms in the present study was rather high, and the level of SDS was substantial (more than one-fifth of the adolescents), the prevalence of CSD was much lower (3.5%) and similar to that reported in other studies on adolescents from primarily North America and Europe [13, 59]. This finding suggests that the combination of the specific symptoms rather than the number of symptoms needs to be taken into account when assessing the prevalence of major depression in the general population. While the prevalence of most of the individual depressive symptoms was higher in girls than in boys, the prevalence of CSD did not differ significantly by gender, which seems to contradict previous reports of an increased risk of depression in females during adolescence [34], and which might be potentially explained by a greater proportion of younger adolescents in the present study. At the same time, when the SDS group was included, the prevalence of depressive symptoms became significantly higher in girls, which is in line with previous research.

The importance of focusing on recent symptoms of depression, compared to lifetime symptoms, was emphasized by previous research, which pointed to the role of a current major depression diagnosis for violence outcomes and suggested that much of the aggressive behavior in individuals with mental health problems in general is associated with increased symptomatology [4]. Similar to previous reports with both adults and adolescents [see 33 for a review], the present study demonstrated that adolescents from the general population with a current depressive episode report higher levels of aggression than their non-depressed peers and that these differences involved both cognitive-emotional aspects of aggression (anger rumination and trait anger), but also behavioral aspects, such as physical, verbal and social aggression.

In addition, depressed adolescents also had higher scores on almost all of the comorbid conditions, all of which were also linked to different types of anger and aggression. Indeed, comorbid mental illness and substance use represent the highest risk for violence [4]. At the same time, in connection with this it has been suggested that both PTSD and depression may be independently associated with physical aggression toward others [60], or that depressive symptoms may mediate the link between PTSD and aggressive behaviors [7, 61]. Similarly, it has also been hypothesized that emotional dysregulation, commonly observed in depressed individuals, may represent a common denominator underlying the comorbidity between ADHD and aggression [62]. Finally, the use of alcohol and other substances significantly increases the risk for violent outcomes for several types of severe mental illness, including depression [4], by mitigating the relationship between violence and mental illness and making it easier for a person to commit violent acts.

Considering the potential complexity of the association between depression, its comorbid conditions and different types of aggression, it was important to assess how much difference in aggression would remain in relation to depression, after controlling for comorbidity. The substantial decrease in the significance of this association suggests that a major role was played by comorbidity in this relationship, especially with regard to the behavioral aspects of aggression. However, the fact that the association remained significant for social aggression and anger rumination (albeit substantially attenuated) even after controlling for comorbidity, suggests there may be a more direct link between depression and anger and aggression that is unrelated to comorbid symptoms. While both anger rumination and sadness rumination are related to deficits in cognitive inhibition and the decreased ability to eliminate extraneous negative information from the working memory [33], they represent two distinct concepts and are differently related to aggression [63]. Having said this, as sadness and anger are interwoven and often co-occur [28], it has also been suggested that sadness rumination (i.e. the internal attribution of negative events), which is indicative of depression, is closely associated with and can transform into anger rumination (i.e. the external attribution of negative events), which in turn is a precursor to aggression [26].

Previous research on the association between depression and different aspects of aggression by gender has been rather limited and produced inconsistent results. Similar to this study, a number of studies have reported the association being stronger in depressed adult males, as compared to females, with greater hostility [64], impulsivity, irritability and more frequent anger attacks [40], and rumination, being more strongly associated with both depression and physical-verbal aggression in adolescent boys rather than girls [65]. At the same time, other studies have reported that the association between depression and physical aggression may be stronger in both adolescent [11] and adult females [41]. With regard to the gender-specific differences in aggression observed in the present study, boys had higher ratings than girls for physical and verbal aggression, but had lower scores on anger rumination. Some studies have also noted that greater irritability and anger following depressive episodes in males may be associated with higher rates of comorbid problems, such as substance abuse and hyperactive behaviors [40], which was partially supported by the present study, where differences in social aggression between boys and girls became significant after controlling for comorbidity, with higher levels in boys. An interaction effect of depression by gender was also found on social and verbal aggression and anger rumination, with higher scores in depressed boys, suggesting that current depression may increase the risk for these aggression factors in boys even after adjusting for comorbidity.

The present study had several important strengths, including the use of a large community-based sample of adolescents from a non-Western country, use of a clear definition of depressive episode and being able to assess different types of aggression and anger. However, it also had a number of limitations. Assessment was based solely on self-reports, which may have been subject to reporting bias. In addition, studies relying on symptom scales rather than DSM/ICD criteria to identify individuals with major depression are likely to overestimate prevalence rates [66], and the same limitations can be equally applied even to the measures used for assessing comorbidity, making the findings more difficult to replicate, and hence a more clinically sound evaluation with other measures, such as a structured diagnostic interview should be included in future studies. Other types of events or symptoms that may have occurred (and could have impacted on anger and aggression) were not assessed in this study. As this study was cross-sectional in nature, any potential causality could not be established for the observed associations, and future studies should use a longitudinal study design in order to address this issue. The effect size for the interaction terms, especially for verbal aggression, was relatively small, suggesting that the specific impact of the interaction between these factors may be limited. It should also be noted that the hyperactivity/inattention scale of the SDQ had a low internal consistency, likely due to the use of a few, broad items, and reverse scoring, which could have impacted the results. Finally, the exclusion of subjects due to missing data could have resulted in selection bias that may have also affected the results.

## Conclusions and clinical implications

To conclude, the prevalence of CSD (corresponding to a current major depressive episode) in Russian adolescents was similar to that reported for other countries. Most of the overtly aggressive behavior in adolescents associated with depression may be related to its comorbid problems, but depressive symptoms also have an independent association with the cognitive-emotional and covert aspects of aggression, which may also be gender-specific. This study has clinical implications, suggesting that the adequate identification and treatment of depression and its comorbid conditions may have an impact on aggressive behavior in adolescents. As research suggests that the risks for violence outcomes in individuals suffering from depression, but without current depressive symptoms may be substantially lower than in those with such symptoms [4], a focus on more recent symptoms, rather than on the mere presence of a disorder, seems to be particularly relevant when considering the relationship between depression and aggression, especially in the context of recovery-oriented mental health. Finally, studies suggest that other factors may contribute more strongly to violent outcomes for persons with a mental disorder than the mental disorder alone [67, 68], and future research should explore the range of risk-related factors and the interactions between them in order to provide a more nuanced understanding of these complex relationships and to integrate them into sound theoretical models.

## Data Availability

The data used in the study cannot be shared due to the limitations specified in the decision of the ethical committee. All other materials/instruments used in the study can be shared upon request. Please address your requests to the corresponding author (VR).

## References

[1] Stevens H, Laursen TM, Mortensen PB, Agerbo E, Dean K (2015). Post-illness-onset risk of offending across the full spectrum of psychiatric disorders. Psychol Med.

[2] Fazel S, Smith EN, Chang Z, Geddes JR (2018). Risk factors for interpersonal Violence: an umbrella review of meta-analyses. Br J Psychiatry.

[3] Whiting D, Lichtenstein P, Fazel S (2021). Violence and mental disorders: a structured review of associations by individual diagnoses, risk factors, and risk assessment. Lancet Psychiatry.

[4] Van Dorn R, Volavka J, Johnson N (2012). Mental Disorder and Violence: is there a relationship beyond substance use?. Soc Psychiatry Psychiatr Epidemiol.

[5] Krakowski M, Czobor P (2016). Proneness to aggression and its inhibition in schizophrenia: interconnections between personality traits, cognitive function and emotional processing. Schizophr Res.

[6] Graham K, Livingston M (2011). The relationship between alcohol and Violence: population, contextual and individual research approaches. Drug Alc Rev.

[7] Taft CT, Vogt DS, Marshall AD, Panuzio J, Niles BL (2007). Aggression among combat veterans: relationships with combat exposure and symptoms of posttraumatic stress disorder, dysphoria, and anxiety. J Trauma Stress.

[8] Saylor KE, Amann BH (2016). Impulsive aggression as a comorbidity of attention-deficit/hyperactivity disorder in children and adolescents. J Child Adol Psychopharmacol.

[9] Långström N, Sjöstedt GMRV, Fazel S (2009). Risk factors for violent offending in autism spectrum disorder: a national study of hospitalized individuals. J Interpers Viol.

[10] Fazel S, Wolf A, Chang Z, Larsson H, Goodwin GM, Lichtenstein P (2015). Depression and Violence: a Swedish population study. Lancet Psychiatry.

[11] Knox M, King C, Hanna GL, Logan D (2000). Ghaziuddin, N. Aggressive Behavior in clinically depressed adolescents. J Am Acad Child Adol Psychiatry.

[12] Ozkan T, Rocque M, Posick C (2018). Reconsidering the link between depression and crime: a longitudinal assessment. Crim Just Beh.

[13] Costello EJ, Erkanli A, Angold A (2006). Is there an epidemic of child or adolescent depression?. J Child Psychol Psychiatry.

[14] Balazs (2013). Adolescent subthreshold-depression and anxiety: psychopathology, functional impairment and increased Suicide risk. J Child Psychol Psychiatry.

[15] Avenevoli KRC (2001). Merikangas. K. R. Mood disorders in children and adolescents: an epidemiologic perspective. Biol Psychiatry.

[16] Weiss B, Catron T (1994). Specificity of the comorbidity of aggression and depression in children. J Abnorm Child Psychol.

[17] Krakowski M, Nolan K. Depressive symptoms associated with aggression. Psychiatr Times 34(2) (2017).

[18] Angelakis S, Nixon R (2015). The comorbidity of PTSD and MDD: implications for clinical practice and future research. Behav Change.

[19] Rohde P. Comorbidities with adolescent depression. In: Nolen-Hoeksema S, Hilt LM, editors. Handbook of depression in adolescents. Routledge/Taylor & Francis Group; 2009. pp. 139–77.

[20] Blain-Arcaro C, Vaillancourt T (2017). Longitudinal associations between depression and aggression in children and adolescents. J Abnorm Child Psychol.

[21] Afzali MH, Oleary-Barrett M, Séguin JR, Conrod P (2018). Effect of depressive symptoms on the evolution of neuropsychological functions over the course of adolescence. J Affect Disord.

[22] Krakowski M (2003). Violence and serotonin: influence of impulse control, affect regulation, and social functioning. J Neuropsychiat Clin Neurosci.

[23] Soltys KJHDJMBCM (1995). Reid. J. C. characteristics of anger expression in depressed children. J Am Acad Child Adol Psychiatry.

[24] Maj M, Pirozzi R, Magliano L, Bartoli L (2003). Agitated depression in bipolar I disorder: prevalence, phenomenology, and outcome. Am J Psychiatry.

[25] Brennan PA, Mednick SA, Hodgins S (2000). Major mental disorders and criminal Violence in a Danish birth cohort. Arch Gen Psychiatry.

[26] Peled M, Moretti MM (2010). Ruminating on rumination: are rumination on anger and sadness differentially related to aggression and depressed mood?. J Psychopath Behav Assess.

[27] Sukhodolsky DG, Golub A, Cromwell EN (2001). Development and validation of the anger rumination scale. Pers Ind Diff.

[28] Vansteelandt K, van Mechelen I (2006). Individual differences in anger and sadness: in pursuit of active situational features and psychological processes. J Pers.

[29] Spielberger CD. State-trait anger expression inventory: Professional manual. Psychological Assessment Resources, Inc.; 1996.

[30] Besharat MA, Nia ME, Farahani H (2013). Anger and major depressive disorder: the mediating role of emotion regulation and anger rumination. Asian J Psychiatry.

[31] Orth U, Wieland E (2006). Anger, hostility, and posttraumatic stress disorder in trauma-exposed adults: a meta-analysis. J Consult Clin Psychol.

[32] Galen BR, Underwood MK (1997). A developmental investigation of social aggression among children. Dev Psychol.

[33] Dutton DG, Karakanta C (2013). Depression as a risk marker for aggression: a critical review. Aggr Viol Behav.

[34] Breslau J, Gilman SE, Stein BD, Ruder T, Gmelin T, Miller E (2017). Sex differences in recent first-onset depression in an epidemiological sample of adolescents. Transl Psychiatry.

[35] Karriker-Jaffe KJ, Foshee VA, Ennett ST, Suchindran C (2008). The development of aggression during adolescence: sex differences in trajectories of physical and social aggression among youth in rural areas. J Abnorm Child Psychol.

[36] Potegal M, Archer J (2004). Sex differences in childhood anger and aggression. Child Adol Psychiatr Clin North Am.

[37] Lansford JE (2012). Boys’ and girls’ relational and physical aggression in nine countries. Aggr Behav.

[38] Heilbron N, Prinstein MJ (2008). A review and reconceptualization of social aggression: adaptive and maladaptive correlates. Clin Child Fam Psychol Rev.

[39] Painuly N, Sharan P, Mattoo KS (2005). Relationship of anger and anger Attacks with depression: a brief review. Eur Arch Psychiatry Clin Neurosci.

[40] Winkler D, Pjrek E, Kasper S (2005). Anger Attacks in depression–evidence for a male depressive syndrome. Psychother Psychosom.

[41] Posick C, Farrell A, Swatt ML (2013). Do boys fight and girls cut? A general strain theory approach to gender and deviance. Deviant Beh.

[42] Russian Federal State Statistics Service. (2011). “Всероссийская перепись населения 2010 года. Том 1” [2010 All-Russian Population Census, vol. 1]. (in Russian). Federal State Statistics Service. Retrieved from: http://www.gks.ru/free_doc/new_site/perepis2010/croc/perepis_itogi1612.htm, accessed on February 27th, 2022.

[43] Radloff LS, The CES-D, Scale (1977). A self-report depression scale for research in the general population. Appl Psychol Meas.

[44] Carpenter JS, Andrykowski MA, Wilson J, Hall LA, Rayens MK, Sachs B, Cunningham LL (1998). Psychometrics for two short forms of the Center for Epidemiologic Studies-Depression Scale. Iss Ment Health Nurs.

[45] Ruchkin V, Lorberg B, Koposov R, Schwab-Stone M, Sukhodolsky DG (2008). ADHD symptoms and associated psychopathology in a community sample of adolescents from the European north of Russia. J Atten Disord.

[46] American Psychiatric Association. Diagnostic and statistical manual of mental disorders. 5th ed. APA; 2013.

[47] Kassinove H, Eckhardt CI, Tsytsarev SV (1997). Development of a Russian state–trait anger expression inventory. J Clin Psychol.

[48] Ruchkin V, Schwab-Stone M, Vermeiren R. Social and Health Assessment (SAHA): psychometric development summary. Yale University; 2004.

[49] Isaksson J, Sukhodolsky DG, Koposov R, Stickley A, Ruchkin V (2020). The role of gender in the associations among posttraumatic stress symptoms, anger, and aggression in Russian adolescents. J Trauma Stress.

[50] Pynoos RS (1987). Life threat and posttraumatic stress in school-age children. Arch Gen Psychiatry.

[51] Steinberg AM, Brymer MJ, Decker KB, Pynoos RS (2004). The University of California at Los Angeles post-traumatic stress disorder reaction index. Curr Psychiatry Rep.

[52] Ruchkin VV, Schwab-Stone M, Koposov R, Vermeiren R, Steiner H (2002). Violence exposure, posttraumatic stress, and personality in juvenile delinquents. J Am Acad Child Adolesc Psychiatry.

[53] Johnston LD, Bachman J, O’Malley P. M. Monitoring the future. Institute for Social Research, University of Michigan; 1990.

[54] Goodman R (1997). The strengths and difficulties Questionnaire: a research note. J Child Psychol Psychiatry.

[55] Achenbach TM, Rescorla LA, Ivanova MY (2012). International epidemiology of child and adolescent psychopathology I: diagnoses, dimensions, and conceptual issues. J Am Acad Child Adol Psychiatry.

[56] Ruchkin V, Koposov R, Schwab-Stone M (2007). The strengths and difficulties Questionnaire: Scale validation with Russian adolescents. J Clin Psychol.

[57] Schwab-Stone M, Koposov R, Vermeiren R, Ruchkin V (2013). Cross-cultural findings on community Violence exposure and internalizing psychopathology: comparing adolescents in the United States, Russia, and Belgium. Child Psychiat Hum Dev.

[58] World Health Organization. (2017). Depression and other common mental disorders: global health estimates. Retrieved from: https://www.who.int/publications/i/item/depression-global-health-estimates, accessed 14 March, 2022.

[59] Polanczyk GV, Salum GA, Sugaya LS, Caye A, Rohde LA (2015). Annual research review: a meta-analysis of the worldwide prevalence of mental disorders in children and adolescents. J Child Psychol Psychiatry.

[60] Angkaw AC, Ross BS, Pittman JO, Kelada AM, Valencerina MA, Baker DG (2013). Post-traumatic stress disorder, depression, and aggression in OEF/OIF veterans. Mil Med.

[61] O’Donnell C, Cook JM, Thompson R, Riley K, Neria Y (2006). Verbal and physical aggression in World War II former prisoners of War: role of posttraumatic stress disorder and depression. J Trauma Stress.

[62] Waltereit R, Giller F, Ehrlich S, Roessner V (2019). Affective dysregulation: a transdiagnostic research concept between ADHD, aggressive behavior conditions and borderline personality traits. Eur Child Adol Psychiatry.

[63] du Pont A, Rhee SH, Corley RP, Hewitt JK, Friedman NP (2018). Rumination and psychopathology: are anger and depressive rumination differentially associated with internalizing and externalizing psychopathology? Clin. Psychol Sci.

[64] Sethi BB, Prakash R, Arora U (1980). Guilt and hostility in depression. Ind J Psychiatry.

[65] Rey L, Extremera N (2012). Physical-verbal aggression and depression in adolescents: the role of cognitive emotion regulation strategies. Universitas Physiol.

[66] Ferrari AJ (2013). Burden of depressive disorders by country, sex, age, and year: findings from the global burden of Disease study 2010. PLoS med.

[67] Swanson JW, Holzer CE, Ganju VK, Jono RT (1990). Violence and psychiatric disorder in the community: evidence from the epidemiologic catchment area surveys. Hosp Community Psychiatr.

[68] Corrigan PW, Watson AC (2005). Findings from the national comorbidity survey on the frequency of violent behavior in individuals with psychiatric disorders. Psychiatry Res.

